# Expression of TNF-related apoptosis-inducing ligand (TRAIL) in keratinocytes mediates apoptotic cell death in allogenic T cells

**DOI:** 10.1186/1750-1164-3-13

**Published:** 2009-11-19

**Authors:** Kerstin Reimers, Christine Radtke, Claudia Y Choi, Christina Allmeling, Susanne Kall, Paul Kiefer, Thomas Muehlberger, Peter M Vogt

**Affiliations:** 1 Department of Plastic, Hand and Reconstructive Surgery, Medical School Hannover Podbielskistraße 380, 30659 Hannover, Germany; 2 University Duesseldorf, Moorenstrasse 5, 40225 Duesseldorf, Germany

## Abstract

The objective of the present study was to evaluate the aptitude of TRAIL gene expression for inducing apoptosis in co-cultivated T-cells. This should allow preparing a strategy for the development of a durable, allogenic skin substitute based on the induction of an immune-privileged transplant. In order to counteract the significant potential of rejection in transplanted allogenic keratinocytes, we created a murine keratinocyte cell line which expressed TRAIL through stable gene transfer. The exogenic protein was localized on the cellular surface and was not found in soluble condition as sTRAIL. Contact to TRAIL expressing cells in co-culture induced cell death in sensitive Jurkat-cells, which was further intensified by lymphocyte activation. This cytotoxic effect is due to the induction of apoptosis. We therefore assume that the de-novo expression of TRAIL in keratinocytes can trigger apoptosis in activated lymphocytes and thus prevent the rejection of keratinocytes in allogenic, immune-privileged transplants.

## Introduction

Members of the TNF ligand family control and conduct numerous immunological and inflammation-related reactions. The Fas-FasL system and its associated mechanism of activation-induced cell death play an important role for the maintenance of hemostasis of the lymphoid system and the induction of immune tolerance [1]. The TNF-related apoptosis-inducing ligand (TRAIL) was identified as a homologue of the Fas-ligand (FasL) [2]. Yet, in contrast to FasL, TRAIL expression has been demonstrated in various tissues and organs [2-4], as the expression pattern of TRAIL receptors allows a subtle observation of apoptotic reactions. Until now, five different receptors for TRAIL have been described and all belong to the TNF receptor family. The receptors TRAIL-R1/DR4, TRAIL-R2/DR5, TRAIL-R3/DcR1 and TRAIL-R4/DcR2 exhibit a considerably homologous sequence of their extracellular domain and bind TRAIL as the only known ligand. The soluble receptor osteoprotegerin (OPG) belongs to a different sub-family and binds the ligand RANKL/OPGL as well. Following ligand binding and activation of cytoplasmatic death domains, TRAIL-R1 and TRAIL-R2 start series of signals for apoptosis [4,5], whereas the decoy receptors do not transmit death signals. The ratio of expression of DR4/DR5 and decoy receptors by a tumor cell will determine its sensibility for TRAIL-induced apoptosis [2,4]. An important function of TRAIL is the regulation of the immune response being involved in controlling the extent of the activated lymhocyte reaction [6]. Interactions between TRAIL and lymphocytes can create so-called immune-privileged sites, e.g. the placenta [7].

The use of TRAIL for the induction of tolerance against allogenic transplants should be considered in burn medicine. The therapy of massive burn injuries is highly complex and can result in grave personal and socio-economic consequences. The therapeutic gold standard is the early resection of necrotic tissue and subsequent wound coverage with autologous skin transplants. Extensive burn injuries are associated with a lack of sufficient donor areas and the need for temporary allogenic or alloplastic coverage. However, allogenic transplants will ultimately be rejected, once the patient regained immunological competence. Allogenic keratinocytes exhibit a profound intrinsic immunogenicity due to the antigen-presenting properties of epidermal cells [8] which express MHC II and a host of inflammatory cytokines. The resulting rejection of the transplant is predominantly T-cell mediated, where even a small number of donor-specific T-cells can lead to the complete destruction of the transplanted skin [9]. Experimental data show that skin allografts are rejected by either CD4^+ ^or CD8^+ ^T cells at any degree of antigenic mismatch [10]. Consequently, the final wound coverage has to consist of autologous split-thickness skin grafts from repeatedly harvested donor sites. The prognosis of the patient can deteriorate due to rising numbers of surgical interventions, immunological reactions against the allotransplants, increasing danger of infection and inadequate wound healing of the donor areas. The production of autologous epidermal cells from full-thickness skin biopsies is hampered by long intervals of cultivation of up to three weeks. The long-term quality and stability of these keratinocyte sheets are inferior to alternative methods of coverage [11].

The development of a storable, cutaneous allo-transplant with a reduced immunogenic reactivity could help to solve most of these problems.

The aim of this study was to evaluate a concept for the induction of tolerance through the genetic transfer of TRAIL cDNA in keratinocytes to produce a localized lymphocytic apoptosis. We investigated whether the transfer of the TRAIL gene in cultivated keratinocytes could be cytotoxic for co-cultivated T-cells. Our results show that the constituitive expression of TRAIL after direct contact between effector and target cells will successfully trigger apoptosis in T-cells and thereby limit the transplant rejection. The use of keratinocytes expressing TRAIL could create an immune-privileged status for the successful transfer of allogenic keratinocytes as coverage of extensive burn wounds.

## Materials and methods

### Cell culture

The murine epithelial cell line JB6 (American Type Culture Collection, Manassas, VA) was cultured in MEM with 10% fetal calf serum (FCS), 2 mM Glutamine and 1% non-essential amino acids (Biochrom, Berlin, Germany) at 37°C and 5% CO_2 _in humidified atmosphere. The human T lymphoblastoma Jurkat cell line was maintained in RPMI supplemented with 10% FCS. For co-culture experiments JB6 cells were plated at a densitiy of 2,5 10^5^/ml in six well tissue culture plates. Jurkat cells were either used untreated or stimulated for two days with ConA (1 μg/ml). Cells were washed and grown co-cultured for 48 hours in RPMI with 10% FCS.

*Reagents *Antibodies directed against the C-terminus of TRAIL were obtained from Santa Cruz Biotechnology, Inc. (Santa Cruz, CA) (clone: D-3) and BD Pharmingen (San Diego, CA) (clone: B5.1). Antibodies directed against the N-terminus of TRAIL were obtained from Chemicon (Temecula, CA). Antibodies directed against Caspase 8 were obtained from Santa Cruz Biotechnology. Secondary antibodies were purchased from Sigma (Taufkirchen, Germany) and Molecular Probes (Leiden, The Netherlands), respectively. Recombinant human TRAIL was obtained from Calbiochem (Schwalbach, Germany).

### Generation of TRAIL producing cell clones

Full length TRAIL cDNA was cloned from a human lymphoma cDNA library (Lym12, Invitrogen, CA) using -ATG GCT ATG ATG GAG GTC- as 5' and -TTA GCC AAC TAA AAA GGC- as 3' primer, according to the published sequence (GeneBank accession number: U37518), and cloned into the expression vector pcDNA5/FRT (Invitrogen). For generating transfected cell lines JB6 pFRT/lac Zeo cells were transfected with the expression vector and pUG44 with the non-liposomal transfection reagent FuGene6 (Roche Molecular Biochemicals, Mannheim) following the supplier's instructions. Recombinant cells were selected with Hygromycin B (Calbiochem). β-galactosidase expression was measured by a specific ELISA obtained by Roche Molecular Biochemicals (Mannheim, Germany) and performed according to instructions and visualized by staining with 5-bromo-4-chloro-3-indolyl-beta-D-galactopyranoside (X-Gal).

### Immunofluorescent staining

Cells were fixed in 4% Paraformaldehyde for twenty minutes at room temperature. Intracellular staining was achieved by treatment with 0.2% Triton-X-100. After blocking in 2% FCS/PBS cells were incubated with primary antibodies followed by secondary antibodies coupled to Alexa 488 or Alexa 546, respectively, at 37°C for 1 hour. Cells were covered with vector shield (Molecular probes) and examined by immunofluorescence microscopy.

### Western Blot Analysis

Cells were harvested by centrifugation at 300 g, the pellet was resuspended in 100 μl RIPA buffer (10 mM Tris, pH8, 150 mM NaCl, 1% Nonidet P-40, 0.5% sodium desoxycholate, 0.1% sodium dodecyl sulfate (SDS), 1 mM phenylmethylsulfonyl fluoride, 4 μg/ml aprotinine, 1 mM sodium orthovanadate), sonicated and spun at 16600 g for 5 min to remove insoluble material. The protein content was determined by Bradford assay. Samples were mixed with Laemmli buffer and subjected to SDS-polyacrylamide gel electrophoresis followed by Western blotting to nitrocellulose membrane using standard techniques. Filters were blocked in 5% non-fat dried milk supplemented with 0.05% Tween 20. Immunodetection was performed either for 1 hour at room temperature or at 4°C over night with the antibodies of interest. NBT/BCIP (Roche) was used as a substrate to detect bound antibody.

### Detection of cell death

FITC-Annexin V (Roche) staining was performed according to the manufacturer's instructions. After staining the cells were examined by immunofluorescence. Quantification of apoptotic cells was done with Apo-One assay (Promega) as recommended by the manufacturer. In brief, Jurkat cells were harvested from the co-cultures after 48 hours. 10000 cells were seeded into 96 well plates in RPMI medium. 100 μl substrate reagent was added and incubated at 37°C for one hour. Fluorescence was measured at 485 nm with a fluorescence reader (Genios, Tecan, Austria).

### Proliferation assays

To quantify proliferation commercial kits (Cell titre blue, Promega) were used. 10000 Jurkat cells were seeded into 96 well plates. Substrate was added according to the manufacturer's instructions. Incubation was done at 37°C for one hour. The plates were read out at 590 nm.

*ELISA *To detect soluble TRAIL in cell culture medium ELISA (Biosource) was performed. Supernatants were collected from cells after 48 hours of cultivation. Adherent cells were lysed. Protein concentrations were determined using Bradford assay and normalised. Samples were added to precoated 96 well plates and ELISA was carried out as recommended by the manufacturer.

### Statistics

Data were collected from at least three independent experiments and are presented as the mean and SEM. The significance was determined by student's T test.

## Results

### Generating a keratinocyte cell-line to express TRAIL

The present study investigated the capacity of overexpressed TRAIL to induce apoptosis in co-cultivated T-cells. In order to achieve higher reproducibility and standardization of results, we used stably transfected cells. A host cell line was established integrating a stable gene expression cassette with a lacZ-zeocin fusion gene. The hybrid gene was transcribed under the constitutive CMV promoter. However, the efficacy of the transcription may vary depending on the genomic insertion point of the integrated gene box. As a precaution, we used genetically identical cell clones to determine the rate of lacZ expression.

**Figure 1:**
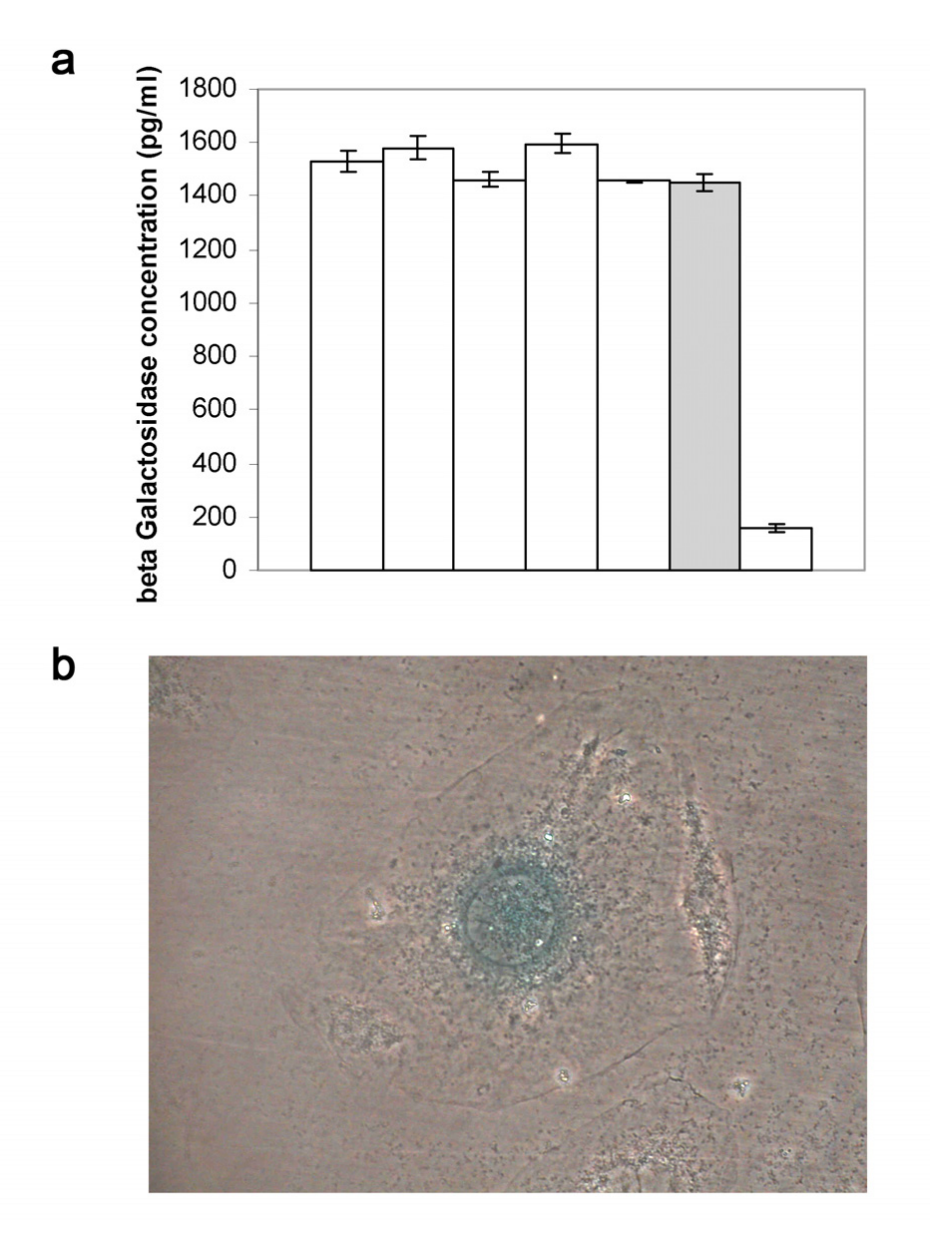
**LacZ expression in the JB6 host cell line**. The murine keratinocyte cell line JB6 was transfected with a FRT flanked lacZ-Zeocin expression cassette. Clone JB6-pFRTLACZeo-7 was chosen for single genomic integration of the cassette and high β-galactosidase expression. a ELISA measurement of the rate of expression of β-galactosidase in chosen cell clones. Clone JB6-pFRTLACZeo-7 is indicated as a grey bar. b Formaldehyde fixated JB6-pFRTLACZeo-7 cells dyed with X-Gal.

Fig 1b depicts a murine keratinocyte derived from clone JB6-pFRTLACZeo-7, which was incubated with the galactose equivalent X-Gal following fixation with formaldehyde. The rate of expression of β-galactosidase was ascertained with ELISA measurements (fig 1a). The clones exhibiting the highest expression of β-galactosidase were examined for the genomic integration of the gene box with Southern Blot technique (fig 1c). Clone 7 showed not the highest rates of expression. Yet, it was selected as the host cell line because the integration of a single gene box promised higher uniformity for the further course of the study.

The lacZ-zeocin cassette flanked by FRT sites can be exchanged for another FRT flanked gene box, if a Flp recombinase derived from S. cerevisae is co-expressed. The insertion of the entire cDNA for TRAIl was intended. The complete open reading frame for TRAIL was amplified from a human B-cell lymphoma cDNA library with specific primers developed from the published sequence (Accession number: U37518). It was then cloned into the vector pcDNA5/FRT and checked by sequencing. The resulting expression vector was used to examine whether the recombinant expression of TRAIL in keratinocytes could trigger apoptosis in co-cultivated T-cells.

### Transfected cells express TRAIL as a functional membrane protein

**Figure 2:**
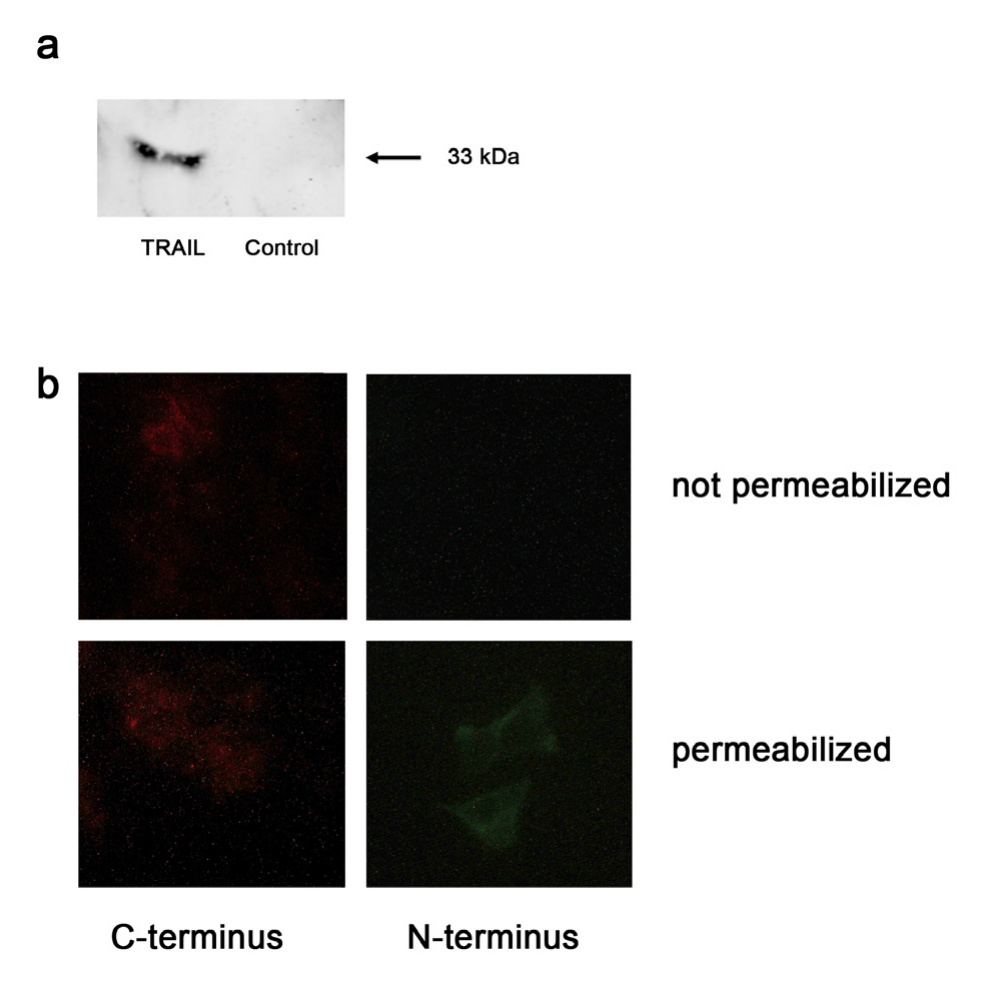
**TRAIL expression of cell line JB6-pcDNA5/FRT TRAIL**. The lacZeo expression cassette was exchanged for the full-length cDNA of TRAIL by FRT recombination. The resulting cell clone was examined for TRAIL expression. a Western Blot of the cellular lysates of JB6pcDNA5/FRT TRAIL and JB6pcDNA5/FRT CAT. Immunostaining was performed by treatment with anti-TRAIL followed by incubation with alkaline phosphatase conjugated secondary antibody and colour development of NBT/BCIP. b Indirect immunofluorescence of JB6pcDNA5/FRT TRAIL. Cells were fixated and incubated with anti-TRAIL either directed against the C terminus or directed against the N terminus. The intracellular domain is detected only if the cells are permeable.

TRAIL has been identified as a homologue member of the TNF family and represents a type II transmembrane protein [2]. It can exist in a soluble condition, which can either be secreted in micro-vesicles [12] or split off the cellular surface [13]. In order to demonstrate that JB6 cells can express TRAIL constitutively as a functional integral membrane protein, they were transfected with an expression box of human TRAIL cDNA. The cell clone JB6pcDNA5/FRT TRAIL resulted from transfection of the murine host cell line JB6pFRTlacZeo clone 7 with the constructed TRAIL vector and the cDNA for a Flp recombinase (pUG44, Invitrogen) followed by a hygromycin selection for recombinants. This particular cell clone was negative for the expression of lacZ and zeocin, but postive for the expression of TRAIL. Using Western Blot technique with TRAIL specific mabs, a 33 kDa band was detected in the total cell lysate of JB6pcDNA5/FRT TRAIL cells (fig 2a). This finding is equivalent to the size of membrane-bound TRAIL. Cells, which had a bacterial CAT gene inserted, instead of the TRAIL gene, did not exhibit a corresponding band. Thus, an expression of endogenic TRAIL was not demonstrated in these cells.

Immunofluorescence microscopy was conducted to show the localization of the gene products expressed (fig 2b). JB6pcDNA5/FRT TRAIL cells exhibited a strong membrane-bound fluorescence which, in contrast, was absent in the control groups of non-transfected or CAT transfected cells. The right orientation of the exogenous protein was confirmed by two different antibodies binding to the N-terminus and the C-terminus only. It showed the cytoplasmic N-terminus could only be detected when the cells were permeabilised with Triton-X-100 (fig 2b).

### Jurkat cell co-cultivated with keratinocytes expressing TRAIL undergo increased cell death

TRAIL is cytotoxic for Jurkat cells through binding on the TRAIL receptor TRAIL R2/DR5. In order to demonstrate the functional activity of TRAIL expressed by the cell clone JB6, Jurkat cells sensitive to TRAIL [14] were placed in co-culture with the cell clone. The survival rate of the Jurkat cells was examined using an MTT proliferation assay. Apoptosis was measured using a fluorescent caspase substrat assay Apo-One (Promega). The percentage of dead Jurkat cells was calculated from three independent experiments.

**Figure 3:**
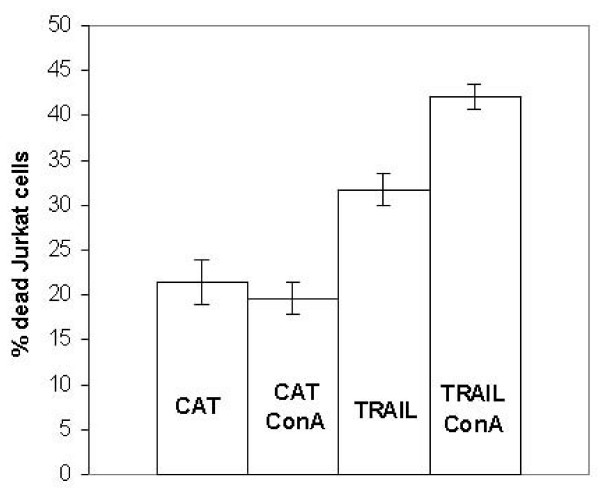
**Percentage of dead Jurkat cells after 48 hrs in co-culture with JB6pcDNA5/FRT TRAIL and CAT, respectively**. Cells were either prestimulated with concanavaline A or left untreated. Cell viabiliy was measured by MTT assay while apoptosis was determined by the caspase substrat reaction Apo-One and the percentage of dead Jurkat cells was calculated. All experiments were carried out in triplicates at least three independent times and presented as means with standard deviation. (p < 0,05 for the difference between cells expressing CAT and TRAIL, respectively.)

After an incubation period of 48 hrs, the Jurkat cells which coexisted with the keratinocytes expressing TRAIL showed an increased mortality rate when compared with the control group (Fig. 3). Culturing of the supernatant, in contrast, showed no cytotoxic effect on the Jurkat cells, which was in accordance with the results of the ELISA. Co-culturing of Jurkat cells with the cell clone JB6pcDNA5/FRT CAT, with the TRAIL gene replaced by the bacterial CAT gene, did not cause increased cytotoxicity either. In order to prevent a bias through contamination with JB6 cells, we conducted control experiments without Jurkat cells (data not shown). This control series showed no increased levels of cell death. In addition, we determined the amount of cell death found in the JB6 cells in co-culture, which were found to be accordingly low. The results of the co-culture experiments are not due to detached apoptotic JB6 cells. The cell clone produced for our study proved cytotoxic for cells sensitive to TRAIL, which is localized on the cell surface.

Next, the Jurkat cells were stimulated with concanavaline A and placed in the co-cultures. Stimulation with ConA induces an increase of the endogenous expression of TRAIL in T cells [13] and also enhances the autocrine and paracrine sensitivity for apoptosis induced by TRAIL. For this purpose, the cells had been cultured in 1 μg/ml ConA enriched media for 2 days. The cells were then thoroughly washed and added to subconfluent JB6pcDNA5/FRT TRAIL or JB6pcDNA5/FRT CAT, respectively. We found an increase of the paracrine sensitivity for the membrane-bound TRAIL in epithelial cells lines.

### Cell contact of TRAIL transfected keratinocytes and T cells leads to caspase activation and apoptotic cell death

**Figure 4:**
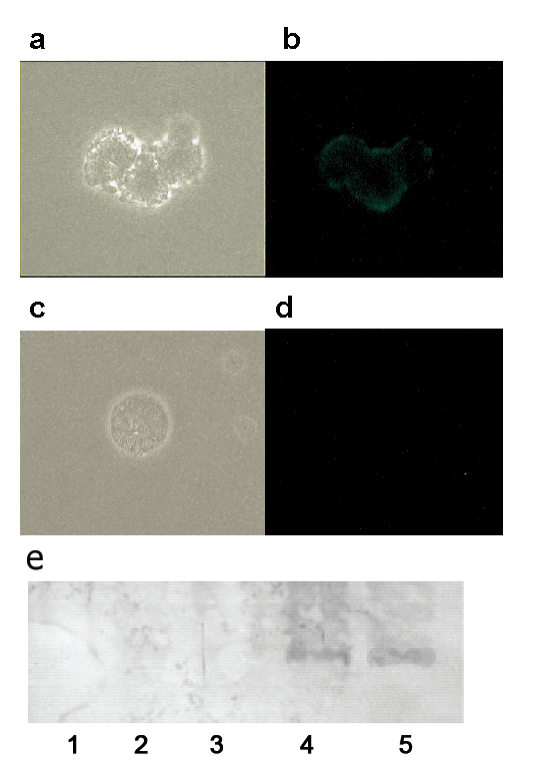
**The effect of TRAIL expression in JB6-pcDNA5/FRT TRAIL cells on co-cultured Jurkat cells**. A Phase-contrast-imaging of apoptotic Jurkat cell after 48 hours of co-culture with JB6-pcDNA5/FRT TRAIL. b Annexin V/Propidiumiodide dyed Jurkat cells after 48 hours of co-culture with JB6-pcDNA5/FRT TRAIL. Membrane fluorescence is characteristic for apoptosis. c Phase-contrast-imaging of a Jurkat cell after 48 hours of co-culture with JB6-pcDNA5/FRT CAT. d Annexin V/Propidiumiodide dyed Jurkat cell after 48 hours of co-culture with JB6-pcDNA5/FRT CAT. No membrane fluorescence could be detected. e Western blot stained with anti-Caspase 8 followed by alkaline phosphatase-conjugated secondary antibody and NBT/BCIP colour development on the blot. Lane 1: untreated Jurkat cells, Lane 2: Jurkat cells kept in co-culture with JB6pcDNA5/FRT CAT, Lane 3: Jurkat cells kept in co-culture with JB6pcDNA5/FRT CAT prestimulated with concanavaline A, Lane 4: Jurkat cells kept in co-culture with JB6pcDNA5/FRT TRAIL, Lane 5: Jurkat cells kept in co-culture with JB6pcDNA5/FRT TRAIL prestimulated with concanavaline A.

Cell death induced by TRAIL shares numerous similar characteristics with other apoptosis promoting factors. Examples are the activation of the caspase cascade, the binding of Annexin V secondary to the shifting of cellular phosphatidylserine and the fragmentation of DNA [2,4,15]. Therefore, we wanted to investigate whether the increased mortality rate of cells in co-culture with JB6pcDNA5/FRT TRAIL was due to a triggered programmed cell death. Phase contrast microscopy of co-cultured Jurkat cells demonstrated apoptotic bodies after 48 hrs (fig 4a). Fluorescence microscopy of co-cultured Jurkat T cells using FITC-Annexin V/propidiumiodide double staining showed that many Jurkat cells in co-culture with JB6pcDNA5/FRT TRAIL were positive for FITC-Annexin V after 48 hrs of incubation (fig 4b). Contamination of the JB6 cells was excluded through measurement of cell size. In the control group JB6pcDNA5/FRT CAT was placed in co-culture with Jurkat cells. These control co-cultures exhibited only very few apoptotic or necrotic cells (fig 4c, d). Protein analysis of Jurkat cells kept in co-culture with JB6pcDNA5/FRT TRAIL for 48 hrs showed the activation of caspase 8 (fig 4e, lane 4). The same could be observed for cells previously stimulated with concanavaline A (fig 4e, lane 5). Western blotting of cellular lysates from untreated cells and cells kept in co-culture with JB6pcDNA5/FRT CAT showed no activated caspase 8 (fig 4e, lanes 1-3). It was thus demonstrated that the contact with JB6pcDNA5/FRT TRAIL will trigger the caspase dependent programmed cell death in Jurkat cells.

### Activation of apoptosis depends on the direct contact to TRAIL expressing keratinocytes

**Figure 5:**
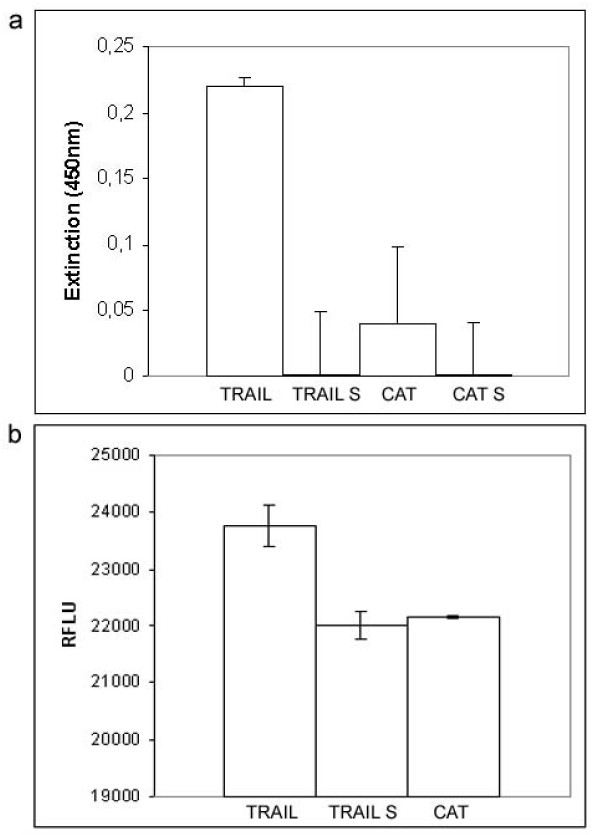
**Influence of the exogenous expressed TRAIL depends on the direct cell contact**. a ELISA quantification of TRAIL. The supernatants of JB6pcDNA5/FRT TRAIL and JB6pcDNA5/FRT CAT cells were collected after 48 hrs of cultivation. The remaining cells were lysed. Protein contents were determined and both fractions were subjected to ELISA specific for TRAIL. b Caspase activity in Jurkat cells kept in co-culture with JB6pcDNA5/FRT TRAIL and JB6pcDNA5/FRT CAT or the harvested cell culture supernatant were determined. Data are reported in relative fluorescence light units (RFLU) and presented as mean with standard deviation of three independent experiments. p < 0,05 was regarded as statistically significant.

In order to determine the amount of membrane-bound TRAIL cleaved by proteases, we performed a TRAIL specific ELISA of the supernatant. Cell culture media of JB6pcDNA5/FRT TRAIL was taken after 48 hrs of incubation and the cells were lysed in 500 μl lysis buffer after removing adherent cells. The protein contents were determined by Bradford assay. TRAIL was quantified in both fractions by ELISA. The result showed the presence of TRAIL in the cellular extract but not in the supernatant, indicating no detectable cleavage of TRAIL (fig 5a).

Further experiments were designed to determine the influence of direct cell contact of JB6pcDNA5/FRT TRAIL on Jurkat cells compared to the supernatant. The cell viability of Jurkat cells was measured by the conversion of resazurin to the fluorescent resurufin (fig 5b). This conversion depends directly on the viability of the cells. Jurkat cells obtained after 48 hrs from a co-culture with JB6pcDNA5/FRT TRAIL showed a decreased viability compared to the untreated control cells. Viability of cells maintained in the supernatant of JB6pcDNA5/FRT TRAIL cells was comparable to the controls.

### TRAIL expressing keratinocytes induce apoptosis in native T cells

**Figure 6:**
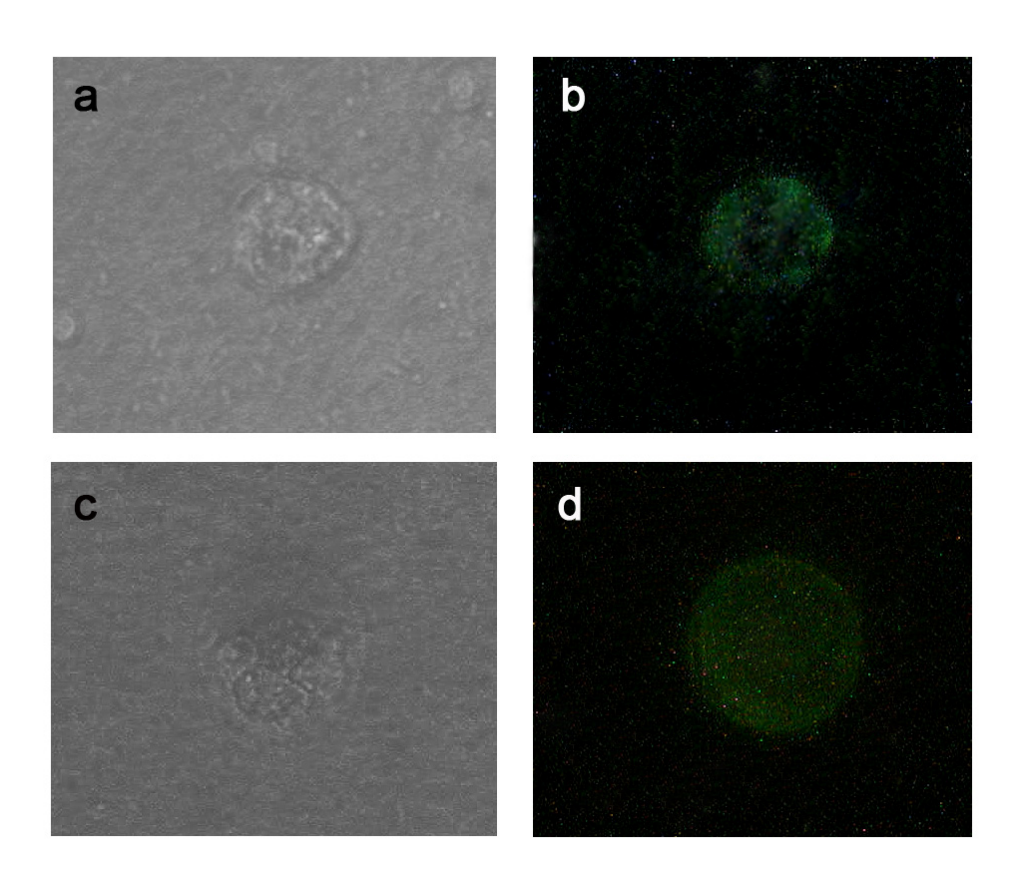
**Co-culturing of JB6pcDNA5/FRT TRAIL with native T cells**. a Phase-contrast-imaging of a apoptotic CD4^+ ^cell after 48 hours of co-culture with JB6-pcDNA5/FRT TRAIL. b Annexin V/Propidiumiodide dyed CD4^+ ^cell after 48 hours of co-culture with JB6-pcDNA5/FRT TRAIL. Membrane fluorescence is characteristic for apoptosis. c Phase-contrast-imaging of a CD8^+ ^cell after 48 hours of co-culture with JB6-pcDNA5/FRT TRAIL. d Annexin V/Propidiumiodide dyed CD8^+ ^cell after 48 hours of co-culture with JB6-pcDNA5/FRT TRAIL.

In order to determine the effect of the co-culture on native T cells, CD4^+ ^and CD8^+ ^cells were isolated from human PBMCs using paramagnetic antibodies. After a 48 hr period of co-culturing, the re-isolated cell populations stained positive with FITC-Annexin V in contrast to cells co-cultured with JB6pcDNA5/FRT CAT (fig 6b, d). Light microscopy revealed characteristic signs of programmed cell death, e.g. membrane blebbing and nuclear condensation (fig 6a, c).

## Discussion

The transplantation of allogenic skin as a live saving therapy after massive burn injuries is an important issue for the application of immuno-modulating strategies. The utilization of apoptosis-inducing properties of some members of the TNF ligand family for the development of immune-privileged transplants is a fascinating, yet highly controversial means of allogenic transplantation. In a mixed lymphocyte-endothelial cell culture model, Cappellesso et al. showed that FasL transfected endothelial cells were effective in inducing apoptosis in Jurkat T cells. The authors conclude that expression of FasL in endothelial cells could be interesting to convey a death signal and induce hyporesponsiveness of allo reactive T cells for organ transplantation [16]. The precise role of the FasL//Fas system for the so-called tumor counter attack has not been clarified yet [17-19]. Various data from in-vitro and in-vivo studies suggest an association between the expression of FasL, which is present in numerous tumours [20-23], and a significant localized reduction of tumor-infiltrating lymphocytes (TILs)[24] secondary to apoptosis of sensitive T-cells [23,25]. FasL acts in an analogous function to establish an immune-privileged status for special tissues, thereby protecting delicate organs like the eye against potential damage from immunological reactions [26]. Similar findings were noticed for TRAIL, which shares numerous characteristics with FasL. The functional expression of TRAIL was demonstrated in human colon adenocarcinoma cell lines [27], possibly representing a biochemical mechanism of tumor escape in analogy to the FasL expression. FasL and TRAIL conjointly contribute to the immune-privileged status of the placenta [7]. In order to evaluate the potential capability of TRAIL gene expression to induce tolerance through the initiation of apoptosis in a localized T cell population, we produced a keratinocyte clone expressing TRAIL which would trigger apoptosis in co-cultured Jurkat cells.

We showed how the stable transfection of a complete cDNA of TRAIL with a strong, constituitive promoter will lead to a lasting expression of membrane-bound, functional TRAIL on the cell surface of the transfected keratinocyte cell line. Jurkat cells are sensitive for the cytotoxicity mediated by TRAIL because of the expression of death-inducing receptors like TRAIL-R2/DR5 [2,4]. Inoue et al. showed how apoptosis in Jurkat cells can be triggered by tumor cells using a TRAIL dependant signal pathway [27]. In analogy to FasL, TRAIL can be expressed in many different tumor cells, ranging from breast- and braintumors to myeloid, lymphoid and colon carcinoma cells. Although the significance of FasL and TRAIL for the establishment of tumors has not been clarified in vivo, the induction of apoptosis represents an effective mechanism for the selective limitation of TILs, which can possibly be applied in transplants. The findings of our study show a pronounced cytotoxicity of complete TRAIL on TRAIL sensitive Jurkat cells and freshly isolated CD4^+ ^and CD8^+ ^cells. The cells will die due to a programmed cell death mediated by caspase. The cytotxic effect on Jurkat cells can be amplified by previous activation and incubation with ConA in a so-called activation induced cell death. The cell clone JB6pcDNA5/FRT TRAIL can induce apoptosis in T cells on direct contact and can thus effect a localized reduction of the total number of T cells.

There was no detectable secretion of sTRAIL in the cell culture media with the culturing conditions chosen in this study. The exposure of TRAIL sensitive Jurkat cells to the cell culture supernatant did not produce a cytotoxic effect, however, direct cell contact in the co-culture led to apoptosis in the target cells. An increase of the content of sTRAIL in humans can possibly produce hepatotoxicity and must be carefully considered before the application of specific gene therapy [28,29]. Although TRAIL represents a type II transmembrane-protein, it can also exist in a soluble condition following processing and it will exhibit tumoricidal activity [15,30,31]. sTRAIL can mediate apoptotic signals into cells through interaction with the receptors TRAIL-R1/DR4 and TRAIL-R2/DR5. sTRAIL, however, is not as effective as membrane-bound TRAIL [32,33], which is probably due to conformational differences [34,35]. A gene-therapeutic strategy with foreign gene expression in the target cells should be considered as an alternative option in cancer therapy as well as for the induction of immunological tolerance in allogenic transplants.

Further studies using animal models are required to investigate if TRAIL expression in transplanted keratinocytes can induce immunological tolerance. The long-term effects of TRAIL expression on the inflammatory phase of wound healing as well as the interaction with other dermal cells are currently under investigation.

## Competing interests

The authors declare that they have no competing interests.

## Authors' contributions

KR, CR, and CYC participated in the design of the study and writing of the manuscript. KR cloned the vector and generated the stable cell lines, CR performed immunofluorescence experiments and Western Blottings, CYC carried out the cell death detection assays. CA performed the ELISA. SK measured cell proliferation. PK helped with the study design and data analysis. TM participated in writing the manuscript and helped with data analysis. PMV conceived and coordinated the study and participated in evaluation and discussion of the data. All authors read and approved the final manuscript.
